# Extracellular Vesicles in Tumors: A Potential Mediator of Bone Metastasis

**DOI:** 10.3389/fcell.2021.639514

**Published:** 2021-04-01

**Authors:** Shenglong Li, Wei Wang

**Affiliations:** ^1^Department of Bone and Soft Tissue Tumor Surgery, Cancer Hospital of China Medical University, Liaoning Cancer Hospital and Institute, Shenyang, China; ^2^Department of Tissue Engineering, Center of 3D Printing & Organ Manufacturing, School of Fundamental Sciences, China Medical University, Shenyang, China

**Keywords:** extracellular vesicles, osteoblast, osteoclast, bone metastasis, tumor

## Abstract

As one of the most common metastatic sites, bone has a unique microenvironment for the growth and prosperity of metastatic tumor cells. Bone metastasis is a common complication for tumor patients and accounts for 15–20% of systemic metastasis, which is only secondary to lung and liver metastasis. Cancers prone to bone metastasis include lung, breast, and prostate cancer. Extracellular vesicles (EVs) are lipid membrane vesicles released from different cell types. It is clear that EVs are associated with multiple biological phenomena and are crucial for intracellular communication by transporting intracellular substances. Recent studies have implicated EVs in the development of cancer. However, the potential roles of EVs in the pathological exchange of bone cells between tumors and the bone microenvironment remain an emerging area. This review is focused on the role of tumor-derived EVs in bone metastasis and possible regulatory mechanisms.

## Introduction

Malignant tumors are the second leading cause of death worldwide. Distant metastasis of tumor cells is the most common cause of cancer-related death (Krzeszinski and Wan, 2015; Hiraga, 2019; Karayazi Atici et al., 2020). Bone is a major target for cancer metastasis, only secondary to the lung and liver (Yin et al., 2005; Su et al., 2015). Bone has a unique anatomical and physiopathological state that enhances the metastasis of different cancer types, including prostate, breast, lung, and gastric cancer and melanoma (Liu et al., 2020; Ma et al., 2020; Wang et al., 2020). Tumor cells may migrate from their original location to distant skeletal tissues, where they may exhibit accelerated growth and infiltrate surrounding tissues to form distant metastasis, characteristics associated with the heterogeneity of tumors (Zhang et al., 2019; Scimeca et al., 2020). Various physiopathological processes will be induced by the occurrence of affected bones and metastatic tumor cells, such as the adhesion molecules secreted by tumor cells bound to trabecular bone and stromal cells. Additionally, tumor cells can induce angiogenesis and bone absorption factors (Zhang et al., 2019). Once patients develop bone metastasis, there are no effective therapeutic strategies, and the 5-year survival rate of these patients is significantly lower. Moreover, bone metastasis may be accompanied by many complications (e.g., serious bone pain, pathological fracture, hypercalcemia, and spinal cord compression) that affect a patient’s life expectancy and quality of life (Krzeszinski and Wan, 2015; Vicent et al., 2015; Yao et al., 2020).

In recent years, the mechanisms underlying bone metastasis have been a hot research topic, and more and more data support the “seed and soil” theory, which says that tumor cells can only grow in a microenvironment suitable for their growth (Marazzi et al., 2020; Tamura et al., 2020; Yao et al., 2020). Thus, they form metastatic lesions in specific tissues and organs. Primary tumor cells proliferate, invade blood vessels, and form tumor blood vessels. Tumor cells reach each system in the body through the vasculature, proliferate in the microenvironment of specific organs and tissues suitable for their growth, destroy normal tissue structures, and form metastatic lesions. Bone metastasis involves two processes: (1) cancer cells reach bone by a certain pathway; (2) cancer cell survival and growth in the bone. Under normal conditions, the activities of osteoblasts and osteoclasts are balanced (Laranga et al., 2020; Wang et al., 2020). When bone metastasis occurs, this balance is disrupted, and the higher cell activity determines whether the bone metastasis is osteogenic or osteolytic. Osteogenic bone metastasis is the result of the activation of osteoblasts and the inhibition of osteoclasts. Conversely, osteolytic bone metastasis involves the activation of osteoclasts and the inhibition of osteoblasts (Wood and Brown, 2020).

Extracellular vesicles (EVs) are bi-layered membrane vesicles with a diameter of 30–100 nm secreted into the microenvironment via exocytosis by multiple cells. EVs are rich in many components, including cell-specific proteins, lipids, and RNA (i.e., mRNA, miRNA, and other non-coding RNA) (Dai et al., 2020; Yang E. et al., 2020). These vesicles are secreted by multiple cell types (e.g., tumor cells, macrophages, and fibroblasts) and are widely distributed in the blood, urine, ascites, synovial fluid, breast milk, and other body fluids. EVs carry and transmit important signaling molecules that affect the physiological and pathological state of their target cells (Cheng et al., 2020; LeBleu and Kalluri, 2020). Studies have shown that EVs participate in intercellular communication, immune responses, angiogenesis, and tumor cell growth (Yi et al., 2020). They have now been discovered in multiple cell types. Indeed, tumor cell-derived EVs have become a hot research area in the field of cancer (Tamura et al., 2020), and there have been many reports on EVs in cancer. For instance, EVs may release self-carrying cytokines to enhance the occurrence, development, proliferation, and migration of tumors and make tumors drug-resistant (Vasconcelos et al., 2019; Wortzel et al., 2019). In addition, the anticancer activity of miR-124 delivered by BM-MSC-associated EVs can act on the proliferation, epithelial-mesenchymal transition, and chemotherapy sensitivity of pancreatic cancer cells (Xu Y. et al., 2020). Endometrial cancer cells can promote M2-like macrophage polarization by delivering exosomal miRNA-21 under hypoxia conditions (Xiao et al., 2020). Furthermore, exosomal miR-92a-3p derived from highly metastatic cancer cells promotes the epithelial-mesenchymal transition and the metastasis of low-metastatic cancer cells by regulating the PTEN/Akt pathway in hepatocellular carcinoma (Yang B. et al., 2020). Moreover, neuroblastoma-secreted EVs carrying miR-375 promote osteogenic differentiation of bone marrow mesenchymal stromal cells (Colletti et al., 2020).

In recent years, many studies have demonstrated that tumor-derived EVs are an important component of the microenvironment of bone tumors. This article reviews the roles of tumor-derived EVs in bone metastasis and their potential molecular mechanisms and provides new insights for inhibiting bone metastasis.

## Generation, Composition, and Major Biological Functions of Extracellular Vesicles

The formation of EVs mainly involves four processes, including sprouting, invagination, multivesicular body formation, and secretion (Mathew et al., 2020; Zhang Y. et al., 2020). EVs are generated in endosomes. Invagination of the cell membrane results in the formation of early endosomes, and late endosomes sprout inward to form luminal vesicles that are transformed into multivesicular bodies (MVBs) with multiple small vesicles. After the MVBs fuse with the cell membrane, the inner vesicles sink again, and granular vesicles sprout and are released out of the cell as EVs (Li et al., 2020; Naseri et al., 2020). Upon reaching recipient cells, the EVs release their contents in specific cells through ligand binding, phagocytosis, and fusing with the cell membrane to change the physiological state and function of cells (Huyan et al., 2020).

**FIGURE 1:**
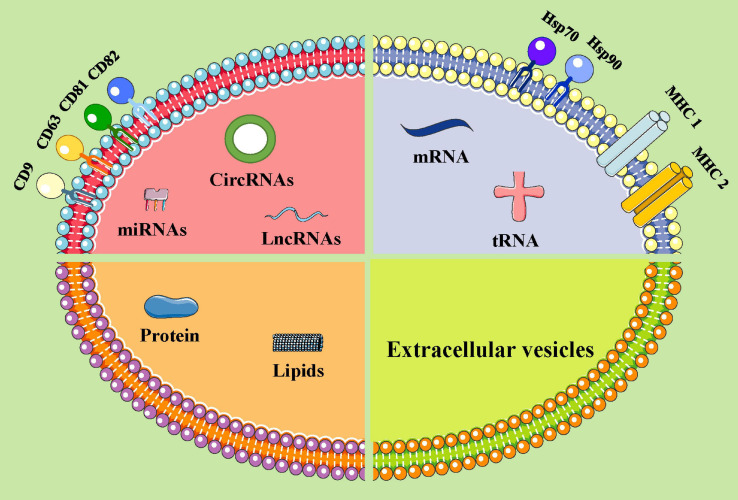
The components of extracellular vesicles (ideograph). EVs can be secreted by various types of cells. EVs carry a variety of proteins, lipids, DNA, mRNA, and non-coding RNAs. Moreover, EV surface proteins contain the following substances: MHC class I/II molecules, heat shock proteins (HSP70, HSP90), and four transmembrane family proteins (CD63, CD9, CD81, and CD82, etc.). miRNAs, microRNAs; lncRNAs, long non-coding RNAs; circRNAs, circular RNAs; mRNAs, messenger RNA; tRNA, transfer RNA.

Extracellular vesicles are mainly composed of proteins, nucleic acids, and lipids (Kalluri and LeBleu, 2020; Zhao X. et al., 2020). All EVs express membrane-bound proteins, such as tetraspanin (CD9, CD63, CD81, and CD82), which may be used as biomarkers and are associated with the biogenetic derivation of EVs (Mathieu et al., 2019; Pegtel and Gould, 2019). EVs derived from different cells express specific proteins, such as ALG-2 interacting protein X (ALix), Wilms tumor type 101 protein (TSG101), and heat shock protein HSP70, and are associated with specific cell functions. EVs also include nucleic acids, such as mRNA, miRNA, and lncRNA (Mathew et al., 2020; Zhang Y. et al., 2020). These nucleic acids fuse with target cells and regulate protein expression and signaling in recipient cells. The lipids in the EVs include cholesterol, diacylglycerol, and phospholipid (Mathew et al., 2020; Zhang Y. et al., 2020). The lipids not only participate in the formation and maintenance of EV morphology but also participate in intercellular communication as signal molecules. These contents are transported to target cells via body fluids and are implicated in angiopoiesis and the occurrence, development, and metastasis of tumors (Kalluri and LeBleu, 2020; Slomka et al., 2020; Zhao X. et al., 2020; Zhang Y. et al., 2020; Figure 1). Potential exosomal biomarkers and clinical targets are displayed in Table 1.

**TABLE 1:** Potential exosomal biomarker and targets in a clinical setting.

**Cargo**	**Type**	**Signaling pathway**	**EVs function**	**References**
TGFβ1	Protein	Activating ERK, Akt, and anti-apoptotic pathway	Promotes tumor growth	Raimondo et al., 2015
*E*-cadherin	Protein	Activating β-catenin and NF-kB signaling pathway	Angiogenesis	Tang et al., 2018
miR-23a	miRNA	Targeted to ZO1	Increases vascular permeability and cancer migration	Hsu et al., 2017
miRNA-191, miR-21, miR-451a	miRNAs	/	Biomarkers for pancreatic cancer	Goto et al., 2018
miR-17-5p, miR-92a-3p	miRNAs	/	Biomarkers for colon cancer	Fu et al., 2018
Glypican-1	Protein	/	Biomarkers for pancreatic cancer	Melo et al., 2015
MET	Protein	Activating MET signaling	Priming premetastatic niches	Peinado et al., 2012
miR-9	miRNA	/	Increasing cancer growth	Baroni et al., 2016
miR-21	miRNA	Regulating PTEN/PI3K/AKT pathway	Inhibits apoptosis and increase drug resistance	Zheng et al., 2017
miR-105, miR-181c, miR-200	miRNAs	/	Increases metastasis	Le et al., 2014; Zhou et al., 2014; Tominaga et al., 2015
ZFAS1	lncRNA	Regulating MAPK signal and EMT	Increases cancer growth and metastasis	Pan et al., 2017

Extracellular vesicles are important carriers for intercellular communication, immunoregulation, and disease diagnosis and prognostic markers (Melo et al., 2015; Tang et al., 2018). Circulating EVs can be used as non-invasive biomarkers and liquid biopsies for early detection, diagnosis, and treatment of cancer (Kalluri, 2016; Hsu et al., 2017; Pan et al., 2017; Fu et al., 2018; Goto et al., 2018). For example, EVs derived from chronic myelogenous leukemia cells contain the cytokine TGFβ1, which binds to the TGFβ1 receptor on leukemia cells, thereby promoting tumor growth by activating ERK, Akt, and anti-apoptotic pathways (Raimondo et al., 2015). Many tumor types are dependent on mitochondrial metabolism by triggering adaptive mechanisms to optimize their oxidative phosphorylation with respect to substrate supply and energy demands. Exogenous EVs may induce metabolic reprogramming through the recovery of cancer cell respiration and the suppression of tumor growth (Tomasetti et al., 2017). Lugini et al. (2016) found that EVs from human colorectal cancer induce a tumor-like behavior in colonic mesenchymal stromal cells, which may be involved in cancer progression or interfere with cancer. Cossetti et al. (2014) discovered that somatic RNA is transferred to sperm cells, which can therefore act as the final recipients of somatic cell-derived information.

Exosomal miRs involved in the modulation of cancer metabolism may potentially optimize diagnosis and therapy (Le et al., 2014; Zhou et al., 2014; Tominaga et al., 2015; Baroni et al., 2016; Zheng et al., 2017). Moreover, tumor-derived exosomes (TDEs) are implicated in the formation and progression of different cancer processes, including tumor microenvironment (TME) remodeling, angiogenesis, metastasis, invasion, and drug resistance (Mashouri et al., 2019). In recent years, the study of EVs has become a new direction for further applied research. For glioblastoma patients, an invasive blood sample is used to diagnose and follow the response to therapy. The protein cargo of plasma GBM EVs can be used to detect a tumor, characterize its molecular profile, and tailor treatment (Osti et al., 2018). In addition, high levels of EVs in the plasma of melanoma patients represent a method for clinical management through the expression of CD63 and caveolin-1 (Logozzi et al., 2009). Rodríguez Zorrilla et al. (2019) also found CD63 expression induced by a dramatic reduction in plasmatic EV after surgical treatment in oral squamous cell carcinoma (OSCC) patients. Lower plasma EV levels correlated with a better life expectancy in OSCC patients (Rodríguez Zorrilla et al., 2019). Expression of both CD81 and PSA are high only in prostate cancer patients, and the levels of tumor biomarkers (e.g., PSA-EVs) may represent a prostate cancer diagnosis (Logozzi et al., 2017). Logozzi et al. (2019a) found that plasma EVs expressing PSA (Exo-PSA) could distinguish prostate cancer patients from healthy individuals both in specificity and sensitivity (Logozzi et al., 2019a).

## Roles and Mechanisms of the Bone Microenvironment in Bone Metastasis

**FIGURE 2:**
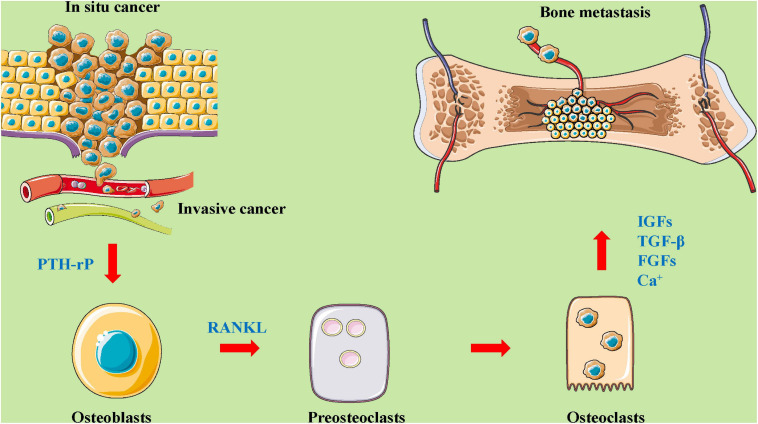
The roles and mechanisms of the bone microenvironment in tumor metastasis to bone. A complex and abnormal cycle of bone metastasis involving mutual interactions between tumor cells, bone cells (osteoclasts and osteoblasts), and the bone matrix. As shown in this figure, *in situ* cancer cells enter the blood vessels, causing proliferation, migration, and invasion. Then, these cells act on osteoblasts by regulating PTH-rP, which affects preosteoclasts and osteoclasts through the mediation of a related protein (such as RANKL). Osteoblasts and osteoclasts affect bone metastases by mediating the expression and secretion of factors (for example, IGFs, TGF-β, FGFs, CA^2+^). FGFs, fibroblast growth factors; IGFs, insulin-like growth factors; PTH-rP, parathyroid hormone-related protein; RANKL, receptor activator for nuclear factor-κB ligand; TGF-β, transforming growth factor-β.

The tumor microenvironment plays an important role in tumor development by establishing interactions between host components and the tumor cells. Factors produced by tumor cells can alter the microenvironment at distant organs, generating pre-metastatic niches for subsequent metastasis. Pre-metastatic niche formation occurs as a sequence of events generated by the tumor cells that effectually prime the target site of disease for arrival, metastasis, and survival. Importantly, the existence of a pre-metastatic niche implies that metastasis to a particular organ is a predetermined event. Endothelial growth factor plays a critical role in the formation of the pre-metastatic niche. The bone microenvironment is rich with different cell types, including osteoblasts, osteoclasts, bone marrow stromal cells, immune cells, and vascular endothelial cells (Browne et al., 2014; Waning and Guise, 2014; Coleman et al., 2020). Osteoblasts and osteoclasts are key cell players in this microenvironment that induce osteolytic, osteogenic, or mixed bone metastasis (Esposito and Kang, 2014; Croucher et al., 2016; Coleman et al., 2020). However, bone marrow stromal cells exert inhibitory effects on bone metastasis through the integrins (αvβ3, α2β1, α4β1), TGFβ family members, bone resident proteins (BSP, OPG, SPARC, OPN), RANKL, and PTHrP (Lipton, 2006; Esposito and Kang, 2014). Moreover, immune surveillance, immune killing, the formation of pre-metastatic lesions, the cooperation of osteoclasts and immune cells, and the bone nutrient supply of vascular endothelial cells are important during bone metastasis (Guise et al., 2006; Quayle et al., 2015; Yao et al., 2020). During this metastatic process, tumor cells interact with cells in the bone microenvironment (Coleman et al., 2020; Mukaida et al., 2020; Figure 2).

Cancer acidity has a major role in inducing increased EV release by human cancer cells. Previous studies demonstrated that low pH was a sign of tumor malignancy that could influence EV release and uptake by human cancer cell lines of different histotypes, particularly prostate cancer (Parolini et al., 2009; Logozzi et al., 2018). Besides, the acquisition of a osteoblastic/osteolytic phenotype, such as CAIX, has been detected both *in vitro*, increased by the low pH condition, and in the plasma of patients, where a clear enzymatic activity, together with a reduced intraluminal pH of EVs was seen (Logozzi et al., 2019b). Tumor acidity and EV levels are strongly related and contribute to the malignant tumor. Buffering, alkalinization, or anti-acidic treatment reduces EV levels in cancer cells. Furthermore, plasma EV levels allow for an early diagnosis of the disease. Therapeutic strategies are being actively pursued to counteract the immunosuppressive and tumor-promoting activities of EVs (Logozzi et al., 2019c).

### Autoregulation of Tumor Cells

The autoregulation of tumor cells is important for determining whether they maintain a latent, dormant state within the bone microenvironment long-term and is a key mechanism against autoimmunity and chemical drugs (Aguirre Ghiso, 2002; Sosa et al., 2014). When tumor cells encounter severe conditions (e.g., hypoxia, hypoglycemia, high acid environment), they may change the modification of proteins in the endoplasmic reticulum. In addition to inhibiting self-proliferation, tumor cells also control their quantity via autophagy (Aguirre Ghiso et al., 1999; Liu et al., 2002). The autophagy of tumor cells is mainly associated with the mammalian target of rapamycin (mTOR) (Malladi et al., 2016). In the case of cellular deficiency, mTORC1 kinase activity is reduced, which induces the autophagy of tumor cells, and they enter into a quiescent period. Tumor cells can also enter a Sox-dependent stem-like state and silence the Wnt signaling pathway, which are important for bone metastasis (Malladi et al., 2016). The autoregulation of tumor cells is a major mode of tumor cell cycle quiescence. When studying the influence of the bone microenvironment on metastatic factors, attention should be paid to the relationship between the bone microenvironment and the intrinsic factors of tumor cells.

### Osteoblasts and Tumor Cells

The interaction between tumor cells and osteoblasts is mainly reflected in the promotion of tumor cell adhesion and colonization by osteoblasts. With continuous research on bone metastasis, more and more experiments have put forth the concept of “pre-metastatic lesions;” that is, after tumor cells reach the target organ, they become infiltrating tumor cells and enter a dormant period (i.e., no proliferation or activation in the short term, Ki67-negative) (Ren et al., 2015; Wang et al., 2018). However, in bone metastasis, the colonization of disseminated tumor cells (DTC) is closely associated with osteoblasts (Grudowska et al., 2017). In addition, Wang et al. (2015) showed that cell activation in early bone metastasis is associated with N–E cadherin and E–E cadherin on the cell membranes of osteoblasts. The binding of osteoblastic *N*-cadherin to *N*-cadherin on tumor cells activates the mTOR signaling pathway to enhance the activation and proliferation of dormant tumor cells. Moreover, osteoblasts are involved in the proliferation process following the activation of the quiescent tumor cells. Thus, osteoblasts participate in a “vicious cycle” between tumor cells and the bone microenvironment, whereby the destruction of the bone microenvironment and tumor cell proliferation promote each other by releasing vascular endothelial growth factor-(VEGF), matrix metalloproteinases (MMPs), thrombospondin, and inflammatory and coagulation factors (Hirshberg et al., 2014; Wang et al., 2015). Recent studies have shown that osteoblasts assist tumor cell colonization through receptors in the early stage of bone metastasis and mainly participate in the “vicious cycle” to promote tumor proliferation in the late stage of bone metastasis (Weilbaecher et al., 2011; Hirshberg et al., 2014).

### Osteoclasts and Tumor Cells

Osteoclasts are an important promoting factor during bone metastasis. At the early stage of bone metastasis, vascular cell adhesion molecule-1 (VCAM1) and the integrin family are important molecules mediating the relationship between tumor cells and osteoclasts (Lu et al., 2011). High VCAM1 levels in tumor cells interact with osteoclast-expressed α4β1 integrin, which may recruit osteoclast precursor cells, initiate the osteoclast process, and ultimately induce the osteolytic clinical manifestations of bone metastasis (Lu et al., 2011). The “vicious cycle” in the bone microenvironment starts with the beginning of the osteoclast process. Current research has demonstrated that osteoclasts are important in bone metastasis and remodeling. As important effector cells, specific inhibition of the tumor-osteoclast process greatly improves the quality of life of patients with bone metastasis (Kelly et al., 2005). The biomarker PTHrP(12–48) can stimulate the expression of cleaved caspase 3 in OCLs and their precursors, causing apoptosis (Kamalakar et al., 2017). PCAT7 is a potential therapeutic target against bone metastasis of PCa via the TGF-β signaling pathway (Lang et al., 2020). Zoledronate enhances osteoclast differentiation through the IL-6/RANKL signal pathway in osteocyte-like MLO-Y4 cells (Kim et al., 2019).

### Bone Marrow Stromal Cells and Tumor Cells

After tumor cells infiltrate the bone marrow, bone marrow stromal cells are important for tumor cell dormancy (Dormady et al., 2000). A recent study indicated that bone marrow stromal cells have an inhibitory effect on tumor cells. It is often necessary to overcome these inhibitory processes to activate dormant tumor cells (Kobayashi et al., 2011). This is also a key target for researchers to design anti-tumor cell drugs to inhibit tumor metastasis and growth.

### Immune Cells and Tumor Cells

An important relationship has been observed between tumor cells as special antigens *in vivo* and autoimmune cells. After tumor cells reach the bone microenvironment, they promote the growth of CD56^+^CD8^+^ T cells and memory CD4^+^ T cells in the bone microenvironment (Feuerer et al., 2001). There is a significant association between CD8^+^ T cells and the incubation period of tumor metastasis as CD8^+^ T cells, as important cells for adaptive immunity, directly kill tumor cells. However, studies have shown that infiltrating dendritic plasma cells continuously release Th2 signals, inhibit CD8^+^ T cells, and promote the maturation of regulatory T cells and dormant tumor growth (Feuerer et al., 2001). A reduction in PDC slows down the activation process for bone metastasis. Depletion of CD8^+^ and CD4^+^ T cells induces tumors to come out of dormancy and apoptosis and induces an increase in tumor cell Ki67, which increases their proliferation and activation (Sawant et al., 2012). Immune cells also participate in the activation of tumor cell dormancy by changing the process of bone remodeling (Sawant et al., 2012). With the rise of tumor immunotherapy, more attention is being paid to bone metastasis and immune cells. However, most of the current bone metastasis animal models are immunodeficient. Researchers should pay attention to the relationship between immune cells and bone metastasis (Kianercy and Pienta, 2016; Wu et al., 2016).

### Vascular Endothelial Cells and Tumor Cells

After tumor cells reach the bone microenvironment, neovascularization becomes an important source of the nutrient supply. Endothelial cells surrounding the neovascularization secrete TGF-β1 and periostin to induce the further proliferation of tumors (Mulcrone et al., 2017). VEGF-α is an important angiogenesis factor that plays an essential role in promoting the activation of tumor cell dormancy (Mulcrone et al., 2017). Nutrient supply is an important resource for cell growth. The study of nutrient supply channels is of great significance for not only bone metastasis but also for tumor metastasis in general and tumor growth *in situ*. In addition, it is essential to study vascular endothelial cells during bone metastasis.

## Extracellular Vesicles and Bone Metastasis

**FIGURE 3:**
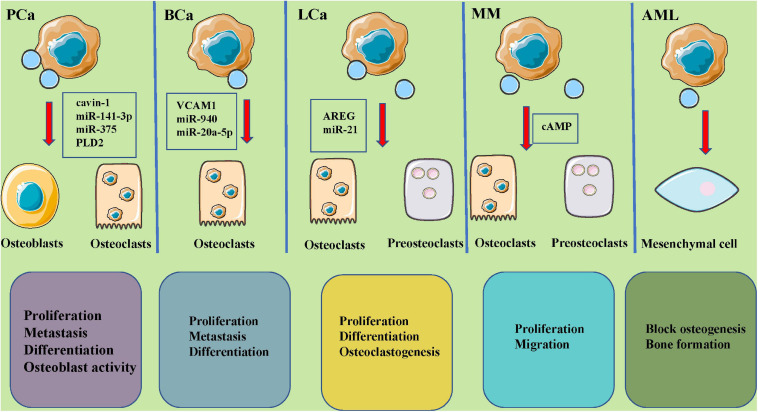
The roles of tumor-derived extracellular vesicles in bone metastases and potential molecular mechanisms. EVs, secreted by PCa (cavin-1, miR-142-3p, miR-375, PLD2), BCa (VCAM1, miR-940, miR-20a-5p), LCa (AREG, miR-21), MM (cAMP), and AML tumors could carry different and abundant content and play a key role in osteoclastic and osteoblastic cycling, leading to various metastatic lesions. PCa, prostate cancer; BCa, breast cancer; LCa, lung cancer; MM, multiple myeloma; AML, acute myelocytic leukemia; TGFB2, transforming growth factor beta 2; AXL, AXL receptor tyrosine kinase; PLD2, phospholipase D2; VCAM1, vascular cell adhesion molecule 1; AREG, amphiregulin.

Recent studies have indicated that tumor-derived EVs play important roles in the microenvironment of bone tumors (Table 2 and Figure 3).

**TABLE 2:** Studies reporting the roles of tumor-derived extracellular vesicles in the development and progression of bone metastases.

**Tumor type**	**References**	**Origin of Evs**	**Isolation of Evs**	**Characterization of Evs**	**Exosomal RNAs**	**How to find**	**Validation of RNAs**	**Target cells**	**Underlying functions**
Prostate cancer	Inder et al., 2014	Prostate cancer PC3 cells	Ultracentrifugation	/	Cavin-1/PTRF	/	Western blot	Osteoblast and osteoclast	Induce osteoblast proliferation, and osteoclast differentiation
	Ye et al., 2017	MDA PCa 2b cells	Ultracentrifugation	TEM, NAT, western blot (CD63, GM130)	miR-141-3p	/	RT-qPCR	Osteoblasts	Promote osteoblastic metastasis
	Li et al., 2019	LNCap cells	Ultracentrifugation	TEM, NTA, western blot (CD9, HSP70, Alix)	miR-375	Small RNA sequencing	RT-qPCR	Osteoblasts (hFOB1.19)	Promote osteoblasts differentiation
	Borel et al., 2020	C4-2B cells	Ultracentrifugation	NTA, TEM, western blot (Alix, CD9, CLN3)	PLD2	/	/	Osteoblasts	Enhance osteoblast activity
Breast cancer	Kang, 2016	Breast cancer cells	/	/	VCAM1	Gene expression profiling	/	Osteoclasts	Mediate the recruitment of pre-osteoclasts and promote their differentiation to mature osteoclasts
	Hashimoto et al., 2018	MDA-MB-231 cell	Ultracentrifugation	NTA, TEM, western blot (CD9, TSG101)	miR-940	Microarray analysis	RT-qPCR	/	Induces extensive osteoblastic lesions
	Guo et al., 2019	MDA-MB-231 cell	Ultracentrifugation	TEM, western blot (CD63, TSG101)	miR-20a-5p/SRCIN1	/	RT-qPCR	Osteoclasts	Enhances the proliferation and differentiation of osteoclasts
Lung cancer	Taverna et al., 2017	CRL-2868 cells	Ultracentrifugation	TEM, western blot (Alix, TSG101, CD63)	AREG	/	/	Osteoclasts	Enhance the proliferation and differentiation of osteoclasts
	Xu et al., 2018	A549 cells	Exosomes isolation kit	TEM, western blot (CD63, TSG101)	miR-21/PDCD4	/	RT-qPCR	Preosteoclasts	Facilitate osteoclastogenesis
Multiple myeloma	Raimondi et al., 2015	Multiple myeloma cells	Ultracentrifugation, sucrose purification	DLS, western blot (Alix, CD63)	/	/	/	Preosteoclast	Modulate pre-osteoclast migration and pro-differentiative role
	Garimella et al., 2014	143B cells	Differential ultracentrifugation	NTA, TEM	cAMP	/	/	Osteoclasts	Contain pro-osteoclastic cargo
Acute myelocytic leukemia	Kumar et al., 2018	AML cells	Ultracentrifugation	NTA, western blot (TSG101, CD63)	Rab27a	/	/	Mesenchymal	Block osteogenesis and bone formation
Melanoma	Mannavola et al., 2019	Melanoma cells	Ultracentrifugation	NTA, TEM, western blot (CD81, TSG101, CANX), flow cytometry	CXCR4/CXCR7	/	RT-qPCR	/	Reprogram the innate osteotropism of melanoma
	Peinado et al., 2012	Peripheral blood of melanoma subjects	Ultracentrifugation, sucrose purification	TEM, western blot (TYRP2, VLA-4, HSP70, HSP90 HSC70)	MET receptor	/	/	/	Induce vascular leakage at pre-metastatic lesions

*CAF, cancer-associated fibroblast; PAF, paracancer-associated fibroblast; AML, acute myeloid leukemia; TEM, transmission electron microscope; NTA, NanoSight NS300 particle size analyzer; DLS, dynamic light scattering.*

### Extracellular Vesicles and Prostatic Cancer Metastasis to Bone

Prostatic cancer is the most common malignant tumor and an important cause of death, with gradually higher incidences and poor efficacies (Archer et al., 2020). Current therapies for prostatic cancer bone metastases continue to focus on reducing symptoms and improving quality of life in these patients. Bone metastases can result in many complications, such as refractory bone pain, hypercalcemia, pathologic fractures, and spinal cord compression, and can even cause more serious complications, such as permanent paralysis (Boucher et al., 2020; Xue et al., 2020). Therefore, an effective treatment strategy to reduce the rate of bone metastases and the associated complications is urgently needed in clinical practice to improve patient survival rates and quality of life.

In recent years, EVs have been reported to be essential for the metastasis of prostatic cancer to bones. Inder et al. (2014) showed that PC3-EVs could induce osteoblast proliferation and osteoclast differentiation. These effects have been shown to be reduced by cavin-1 expression in PC3 cells, which is an important discovery since cavin-1 has also been demonstrated to be a tumor suppressor in caveolin-1-positive prostate cancer. However, a critical question remains, which concerns how cavin-1 expression selectively reduces EV levels in some molecules, including cargo, structural, and functional proteins, and miRNAs. Moreover, Ye et al. (2017) conducted a series of *in vivo* and *in vitro* studies and found that the MDA prostatic carcinoma (PCa) 2b cell-derived exosomal miR-141-3p could transfer from EVs to osteoblasts and promote osteoblastic metastasis by regulating exosomal miR-141-3p levels. These studies indicated that miRNAs could mediate cancer cell-to-osteoblast communication, which is important for the formation of bone metastases and osteogenic damage in PCa. Li et al. (2019) confirmed that high miR-375 expression was observed in LNCap-derived EVs that could preferentially reach osteoblasts and enhance osteoblast miR-375 levels. In addition, exosomal miR-375 might be associated with significantly higher osteoblast activity. Further investigations should be performed concerning the mechanisms underlying bone metastasis in PCa patients, and the molecular mechanisms underlying exosomal miR-375 involvement in the activation and differentiation of osteoblasts. Dai et al. (2019) confirmed that primary PCa cells could educate bone marrow to create a pre-metastatic niche via primary PCa EV-mediated transfer of PKM2 into bone marrow stem cells (BMSCs) with the subsequent upregulation of CXCL12. Furthermore, Borel et al. (2020) showed that phospholipase D2 (PLD2) stimulated EV secretion and enhanced osteoblast activity in PCa cell models. Therefore, PLD2 could be considered a potential player in establishing PCa bone metastases by acting through tumor cell-derived-EVs. EVs, released from prostate tumor cells, decreased DC-STAMP, TRAP, cathepsin K, and MMP-9 expression and thus decreased established markers for osteoclast fusion and differentiation. These findings suggest that tumor cell-derived microvesicles play an important role in cancer progression and disease aggressiveness (Terese et al., 2016).

### Extracellular Vesicles and Breast Cancer Metastasis to Bone

Breast cancer is currently one of the most common malignant tumors in women worldwide, and the incidence of this disease has been rising in recent years. Breast cancer patients develop tumor metastasis during the advanced stages of disease. Bone tissue is one of the most common sites for metastases in patients with advanced breast cancer, and the metastatic ratio is much higher than that of the liver, lung, and kidney (Park et al., 2020; Zhang D. et al., 2020). Breast cancer metastasis to bones not only affects patient quality of life but also results in anemia, fractures, paraplegia, hypercalcemia, pain, cachexia, and increased mortality (Fico and Santamaria-Martinez, 2020). When breast tumors metastasize to bone, the balance between osteoblasts and osteoclasts is damaged. Osteoclasts are continuously activated, as manifested by higher osteoclast activity, resulting in osteolytic diseases with osteolysis and structural bone damage (Zhao Z. et al., 2020). Multiple bone growth factors are activated and released during bone resorption and remodeling, which provide an appropriate microenvironment for tumor cell growth, invasion, and metastasis (Zhao Z. et al., 2020). Once breast cancer cells have migrated to bone, the unique bone microenvironment could help to exchange biological information from the tumor cells to osteoblasts and osteoclasts, breaking the balance between osteolysis or osteogenesis during bone remodeling, which further results in fractures and pain and finally leads to death (Zhang D. et al., 2020; Pang et al., 2021). Although the tumors can be excised in clinical settings, tumor cells have often already spread and metastasized to bone, resulting in osteolytic lesions.

Previous studies have shown that EVs play key roles in breast cancer metastasis to bone. Lim et al. (2011) showed that the transfer of miRNAs from bone marrow stroma to breast cancer cells could involve the dormancy of bone marrow metastases. The results indicated that MDA-MB-231 and T47D breast cancer cells were arrested in the G0 phase of the cell cycle during co-culture with bone marrow stromal cells, and several miRNAs involved in cell proliferation were identified by analyzing miRNA expression profiles. These miRNAs included miR-127, miR-197, miR-222, and miR-223, that target CXCL12. Meanwhile, CXCL12-specific miRNAs were transported from bone marrow stroma to breast cancer cells via gap junctions, resulting in lower CXCL12 levels and reduced cell proliferation. Stroma-derived EVs containing miRNAs also contributed to breast cancer cell quiescence to a lesser degree than miRNAs transmitted via gap junctions. Kang (2016) showed that higher vascular cell adhesion molecule 1 (VCAM1) expression in disseminated breast tumor cells mediated the recruitment of pre-osteoclasts and promoted the differentiation of these cells to mature osteoclasts during bone metastasis formation. Bendinelli et al. (2017) clarified the control of the hepatocyte growth factor/mesenchymal-epithelial transition factor gene (HGF/Met) axis using DNA methylation and found that this axis was significantly involved in the supportive cell-metastatic cell nexus and metastatic outgrowths. This translational research focused on the effects of the microenvironment on breast cancer cell phenotypes and the formation of a pre-metastatic niche, and the colonization of these cells in bone. The results suggested the importance of targeting tumor microenvironments by blocking epigenetic mechanisms that control critical colonization events, such as the HGF/Met axis and the WW domain-containing oxidoreductase (Wwox) gene, and therapies targeting bone metastases. Hashimoto and his colleagues suggested that miRNAs secreted by cancer cells in the bone microenvironment induced bone metastasis phenotypes (Hashimoto et al., 2018). Interestingly, the implantation of miR-940-overexpressing MDA-MB-231 cells induced extensive osteoblastic lesions in the resultant tumors by facilitating osteogenic differentiation of host mesenchymal cells, even though MDA-MB-231 breast cancer cells have commonly been considered an osteolytic-inducing cancer cell line. Tiedemann et al. (2019) identified that extracellular L-plastin and peroxiredoxin 4 (PRDX4) played a specific role in stimulating osteoclastogenesis, thus promoting osteolysis during tumor metastasis to bone, which suggested that information regarding the expression of these proteins might be especially useful in the treatment of cancers that frequently metastasize to bone. Another study demonstrated that miR-20a-5p, transferred from breast cancer cell-derived EVs, enhanced the proliferation and differentiation of osteoclasts by targeting SRCIN1, thereby laying scientific foundations for the development of EV- or miR-20a-5p-targeted therapeutic interventions in patients with breast cancer progression (Guo et al., 2019). Breast cancer cell-derived EVs, associated with the formation of a pre-metastatic niche via the transfer of miR-21 and the regulation of PDCD4 protein levels, play an important role in promoting breast cancer bone metastasis (Yuan et al., 2021). Moreover, breast cancer cell (MDA-MB-231) EVs inhibited osteoblast differentiation and reduced cell numbers and activities by increasing osteoclast formation in a RANKL-independent manner. In osteoblasts, breast cancer cell EVs were shown to enhance transcription and increase angiogenesis and osteoclastogenesis (Loftus et al., 2020).

### Extracellular Vesicles and Lung Cancer Metastasis to Bone

Lung cancer has currently one of the highest incidences of malignancy worldwide. Bone is a distant metastatic site for lung cancer, and the most common sites for lung cancer bone metastases include the spine, chest, and pelvis (Tam et al., 2020). The main routes of bone metastases occur through blood and direct local infiltration with rich blood supplies in the marrow cavity and higher adhesion molecule expression in malignant tumor cells, and a large number of growth factors in bone (Xu S. et al., 2020). After metastatic bone disease, patients develop a series of bone-related events, including pain, hypercalcemia, dyskinesia, spinal cord compression, and pathologic fractures, all of which severely affect patient quality of life (Huang et al., 2020). Taverna et al. (2017) observed that non-small-cell lung cancer (NSCLC) EVs that activated the amphiregulin (AREG)-induced epidermal growth factor (EGFR) pathway in pre-osteoclasts, in turn, caused higher RANKL expression in osteocytes. RANKL could also induce the expression of proteolytic enzymes involved in osteoclastogenesis and thereby trigger a vicious cycle in the process of osteolytic bone metastasis (Taverna et al., 2017). Xu et al. (2018) showed that lung adenocarcinoma-derived exosomal miR-21 showed promise as a facilitator of osteoclastogenesis and thus as a potential therapeutic target of bone metastasis. Understanding new advances in the diagnosis and treatment of lung cancer metastasis to bone holds great significance for prolonging survival and improving therapeutic effects in patients with lung cancer with metastasis to bone.

### Extracellular Vesicles and Multiple Myeloma

Multiple myeloma (MM) is characterized as a proliferation of malignant clonal plasma cells that can infiltrate bone marrow and replace marrow cells. This malignancy also causes the mass production of abnormal immunoglobulins and destruction of bone, producing a series of clinical symptoms and signs that seriously threaten the quality of life and lifespan of middle-aged and elderly adults (Garces et al., 2020; Jeon et al., 2020). At present, the primary therapies include combined chemotherapies and stem cell transplants (Coffey et al., 2019). Since the proliferation ratio of tumor cells and formation of multiple resistant strains is low in MM, the therapeutic efficacy of clinical treatments remains less than ideal. Thus, finding a novel therapeutic target is urgently needed.

There are many factors that cause MM bone disease, high osteoclastic activity is the main reason, although many other factors are involved (Abdi et al., 2013; Rossi et al., 2013; Hameed et al., 2014). The proliferation of MM is markedly related to the increase in the number and activity of osteoclasts. This relationship maintains a balance between the abnormal cycle of bone destruction and tumor cell survival. A report by Raimondi et al. (2015) stated that EVs derived from MM were also able to regulate the migration and differentiation of osteoclasts. Additionally, MM-derived EVs have been shown to have two primary functions. One, it can promote the appearance of osteoclasts, and two, it can help improve bone resorption of mature osteoclast-like cells. Moreover, Garimella et al. (2014) reported that highly invasive and metastatic osteosarcoma 143B cells could produce EVs by using ionomycin and forskolin to actively mobilize intracellular calcium or increase cyclic adenosine monophosphate (cAMP) levels. Raimondo et al. (2019) reported that AREG was specifically enriched in EVs from MM cells and that EV-derived AREG participated in MM-induced osteoclastogenesis by activating EGFR ligands in pre-osteoclasts. The above studies have proven that EVs are the main factors of communication between MM cells and the bone marrow microenvironment. More importantly, the above conclusions support the role of tumor cell-derived microvesicles in disease aggressiveness and cancer progression.

### Extracellular Vesicles and Acute Myelocytic Leukemia

Acute myelocytic leukemia (AML) is a heterogeneous clonal hematopoietic stem cell disease mainly characterized by the severe blockage of myeloid cell differentiation, rapid clonal cell proliferation, and the infiltration of other organs by immature myeloid bone marrow cells (Lu et al., 2017; Zhou et al., 2020). The incidence of AML increases with age. AML is considered the most common acute leukemia in adults, with a low incidence in children, only accounting for 15%∼20% of childhood leukemias (Zhou et al., 2020). Recently, Kumar et al. (2018) analyzed a new AML mouse model and demonstrated the niche transformation model. The study results indicated that the hematopoietic microenvironment could be altered with the help of EV transfer (through the Dickkopf WNT signaling pathway inhibitor 1 (DKK1) gene). It was also found that normal niches could be turned into malignant niches. In addition, mesenchymal progenitor cells increased, and osteogenesis and bone formation were blocked after injecting AML-derived EVs into mice. The proliferation rate of AML cells was also shown to be accelerated by the AML-derived EVs. However, AML-derived EVs could be destroyed by Rab27a targeting, which provided inhibitory effects that could significantly delay the appearance of leukemia (Kumar et al., 2018). In addition, DKK1 was found to belong to a normal hematopoietic and osteogenic inhibitory factor. Therefore, related reports stated that bone cell loss was due to the stimulating effects of AML-derived EVs on DKK1 (Kumar et al., 2018). Thus, targeting EVs could represent a new strategy for cancer treatments. The effective suppression of the hematopoietic microenvironment might be a crucial new direction for the control of malignant blood cell proliferation.

### Extracellular Vesicles and Melanoma Metastasis to Bone

Malignant melanoma (Mm) is a highly malignant melanocyte tumor that accounts for 90% of skin malignancies causing death (Broggini et al., 2020; Mannavola et al., 2020). Mm is formed from the malignant transformation of melanocytes located at the epidermal basement membrane. This tumor is more common on the face and heels and primarily progresses from the malignant transformation of nevi or pigmented spots (Wilson et al., 2020). Mm is highly malignant and vulnerable to lymphatic and blood tract metastases in the early stages. Mm often metastasizes to the liver, lung, kidney, and brain, and a few reports have demonstrated metastasis to the gastrointestinal tract and ovaries. However, metastases to bone marrow have only rarely been reported (Wilson et al., 2020). Mannavola et al. (2019) demonstrated that tumor-derived EVs reprogrammed the innate osteotropism of melanoma cells *in vitro* by upregulating membrane CXCR7. These results provide the possible identification of targets for future drug development to skeletal metastases of malignant melanoma (Mannavola et al., 2019). Peinado et al. (2012) indicated that EVs from highly metastatic melanoma increased the metastatic behaviors of primary tumors by permanently “educating” bone marrow progenitors via the MET receptor. Melanoma-derived EVs also induced vascular leakage in pre-metastatic lesions and reprogrammed bone marrow progenitors toward a c-Kit+Tie2+MET+ pro-vasculogenic phenotype (Peinado et al., 2012). Lower MET expression in EVs weakened the pro-metastatic behaviors of bone marrow cells (Peinado et al., 2012). All of these studies implied that melanoma-derived EVs could facilitate bone metastases using some factors, such as MET.

## Conclusion and Outlook

Bone metastasis, as the major complication in patients with advanced tumors, characterized by osteolytic lesion, often occurring in chest bones. Since metastasized cancer cells can damage bone marrow hematopoiesis and bone structure, bone metastasis becomes the major symptom in advanced diseases, as well as the leading cause of death (Coleman et al., 2020). The destruction of the hemopoietic system by tumor cells mainly results in anemia and an increased infection tendency. Excessive bone growth causes local pain, fracture, and spinal cord compression; even paraplegia can be seen with severe bone damage and not only accelerates the death of patients but also seriously reduces quality of life (Mukaida et al., 2020). The discovery of novel biomarkers and targets to control metastatic bone disease is urgently needed. EVs have been reported to be biomarkers for cancer diagnoses and targets for novel therapies. EVs, as carriers of protein, RNAs, and other bioactive molecules, serve as a tool for cell-to-cell communications and could be effective in regulating the signaling pathways or biological behaviors of recipient cells. This review summarizes the roles of tumor-derived EVs in bone metastasis and concludes that proteins and RNAs in EVs derived from tumor cells could enhance tumor invasion and metastasis and might be useful as targets for cancer therapy and the inhibition of bone metastases.

Although research has proven a vital role for EVs in tumor metastasis to bones, many problems still need to be investigated in future studies. These include the roles of tumor-derived EV compositions (including RNA and DNA) in determining bone-specific metastases, whether tumor metastasis could be prevented by inhibiting the secretion of tumor-derived EVs, especially in bone metastases and other organs that damage bodily functions, and if clinical transformations could be carried out using targeted therapies for tumor metastasis to bone based on discovered molecular mechanisms. Solving these issues will help highlight the underlying mechanisms of bone metastases. Future therapeutic strategies could involve a combination of several drugs that might block multiple targets or pathways simultaneously to improve quality of life, prolong survival, and provide greater therapeutic benefits for patients with bone metastases.

## Author Contributions

SL and WW contributed to original draft preparation, allocation, revision, supplement, and edition. Both authors have read and agreed to the published version of the manuscript.

## Conflict of Interest

The authors declare that the research was conducted in the absence of any commercial or financial relationships that could be construed as a potential conflict of interest.

## Abbreviations

EV: extracellular vesicle
miRNAs: micro RNAs
circRNAs: circular RNAs
lncRNAs: long non-coding RNAs
FGFs: fibroblast growth factors
IGFs: insulin-like growth factors
PTH-rP: parathyroid hormone-related protein
RANKL: receptor activator for nuclear factor- κ B ligand
TGF- β: transforming growth factor- β
DTC: disseminated tumor cells
PCa: prostate cancer
BCa: breast cancer
LCa: lung cancer
MM: multiple myeloma
AML: acute myelocytic leukemia
PLD2: phospholipase D2
VCAM1: vascular cell adhesion molecule 1
AREG: amphiregulin
cAMP: adenosine monophosphate
EGFR: epidermal growth factor receptor ligands
MVB: multivesicular body
TSG101: Wilms tumor type 101 protein
Alix: ALG-2 interacting protein X
TDEs: tumor-derived exosomes
TME: tumor microenvironment
mTOR: mammalian target of rapamycin.
